# A Single Bout of Electroacupuncture Remodels Epigenetic and Transcriptional Changes in Adipose Tissue in Polycystic Ovary Syndrome

**DOI:** 10.1038/s41598-017-17919-5

**Published:** 2018-01-30

**Authors:** Milana Kokosar, Anna Benrick, Alexander Perfilyev, Emma Nilsson, Thomas Källman, Claes Ohlsson, Charlotte Ling, Elisabet Stener-Victorin

**Affiliations:** 1 Department of Physiology, Institute of Neuroscience and Physiology, Sahlgrenska Academy, University of Gothenburg, Gothenburg, Sweden; 2 School of Health and Education, University of Skövde, Skövde, Sweden; 3 Epigenetics and Diabetes, Department of Clinical Sciences, Lund University Diabetes Centre, Lund University, Clinical Research Centre, Scania University Hospital, Malmö, Sweden; 4 Department of Medical Biochemistry and Microbiology, NBIS - National Bioinformatics Infrastructure Sweden, SciLifeLab, Uppsala University, Uppsala, Sweden; 5 Centre for Bone and Arthritis Research, Department of Internal Medicine and Clinical Nutrition, Institute of Medicine, Sahlgrenska Academy, University of Gothenburg, Gothenburg, Sweden; 6 Department of Physiology and Pharmacology, Karolinska Institutet, 17177 Stockholm, Sweden

## Abstract

A single bout of electroacupuncture results in muscle contractions and increased whole body glucose uptake in women with polycystic ovary syndrome (PCOS). Women with PCOS have transcriptional and epigenetic alterations in the adipose tissue and we hypothesized that electroacupuncture induces epigenetic and transcriptional changes to restore metabolic alterations. Twenty-one women with PCOS received a single bout of electroacupuncture, which increased the whole body glucose uptake. In subcutaneous adipose tissue biopsies, we identified treatment-induced expression changes of 2369 genes (*Q* < 0.05) and DNA methylation changes of 7055 individual genes (*Q* = 0.11). The largest increase in expression was observed for *FOSB* (2405%), and the largest decrease for *LOC100128899* (54%). The most enriched pathways included Acute phase response signaling and LXR/RXR activation. The DNA methylation changes ranged from 1–16%, and 407 methylation sites correlated with gene expression. Among genes known to be differentially expressed in PCOS, electroacupuncture reversed the expression of 80 genes, including *PPARγ* and *ADIPOR2*. Changes in the expression of *Nr4a2* and *Junb* are reversed by adrenergic blockers in rats demonstrating that changes in gene expression, in part, is due to activation of the sympathetic nervous system. In conclusion, low-frequency electroacupuncture with muscle contractions remodels epigenetic and transcriptional changes that elicit metabolic improvement.

## Introduction

Polycystic ovary syndrome (PCOS) is a common heritable disorder of unclear etiology. Genetic and epigenetic factors may predispose women to PCOS which is a complex endocrine and metabolic disorder that affects 5–17% of women of reproductive age worldwide^1^. Two hallmarks of this disorder are hyperandrogenism together with hyperinsulinemia^2,3^. Women with PCOS have an increased risk of developing metabolic disturbances and type 2 diabetes^4^. Adipose tissue dysfunction, with enlarged adipocytes and decreased circulating levels of adiponectin, has been demonstrated to be a strong factor in the development of insulin resistance in women with PCOS^5^. Furthermore, there seems to be a strong association between hyperandrogenemia and hyperinsulinemia and both are aggravated by overweight or obesity^6^. Genetics may account for up to 70%, but, in addition, exposure to environmental toxins could modify the epigenome and lead to disruption of the endocrine and metabolic processes in women with PCOS^7,8^. Gene expression regulation is a fundamental process that may affect the adipose tissue phenotype, and it is known that gene expression can be altered by epigenetic changes such as DNA methylation. Studies have recently demonstrated altered DNA methylation and gene expression patterns in subcutaneous adipose tissue from women with PCOS^9,10^. As an example, type 2 diabetes susceptibility gene^11^ *PPARγ*, encoding peroxisome proliferator-activated receptor γ which is a master regulator of adipocyte differentiation and target for insulin-sensitizing drugs^12^, had increased DNA methylation and decreased adipose tissue expression in women with PCOS^9^.

A single bout of exercise improves whole body glucose homeostasis and induces multiple transcriptional and epigenetic changes in skeletal muscle^13^. Six months of regular exercise remodels global methylation changes in subcutaneous adipose tissue from patients with type 2 diabetes^13,14^. A single bout of acupuncture with low-frequency electrical stimulation of needles placed in skeletal muscle and adipose tissue, so-called electroacupuncture, initiates a specific pattern of afferent activity in A-delta and C-fibers^15^. By causing muscle contractions, electroacupuncture activates pathways similar to those activated by muscle contractions during exercise^15,16^. We have demonstrated that a single bout of electroacupuncture increases whole body glucose uptake during euglycemic-hyperinsulinemic clamp experiments and modulates gene and protein expression in skeletal muscles in rats^17^, as well as in overweight and obese women with and without PCOS^18^. The increase in glucose uptake by electroacupuncture was in part mediated via modulation of vagal activity and adipose tissue sympathetic activity^18^.

As a single bout of exercise causes a contraction-induced gene activation, together with dynamic changes in DNA methylation in skeletal muscle^13^, we here aimed to test the hypothesis that a single bout of low-frequency electroacupuncture, with muscle contraction, induces acute and rapid changes in gene expression and DNA methylation in subcutaneous adipose tissue in women with PCOS. To better characterize the adipose tissue of women with PCOS, we also investigated the adipocyte size and concentration of sex steroids in circulation and in adipose tissue and compared with controls matched for age, weight, and body mass index (BMI). In addition, to investigate if changes in gene expression are mediated via activation of the sympathetic nervous system, we tested if blockade of adrenergic receptors in female rats also blocked the activation of selected genes.

## Results

### Clinical Characteristics

**Figure 1:**
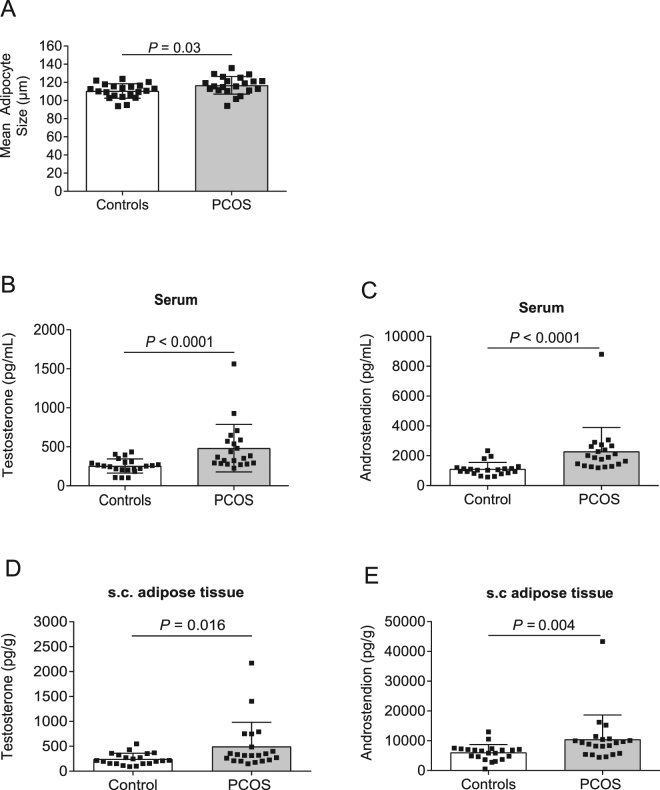
Measurements of adipocyte size. Women with PCOS had larger adipocytes than controls (**A**). Differences in androstenedione and testosterone concentrations in circulation (**B**,**C**), and in subcutaneous adipose tissue (**D**,**E**), between women with PCOS and controls.

For baseline characteristics, 21 women with PCOS and 21 controls matched for age, weight, and BMI were included (Table 1). Thirteen women fulfilled all three diagnostic criteria of PCOS, two presented with hyperandrogenism and ameno-/oligomenorrhea, two presented with hyperandrogenism and PCO morphology, and four presented with ameno-/oligomenorrhea and PCO morphology. Women with PCOS had larger adipocytes than controls (Fig. 1A). Fasting circulating triglycerides and circulating androstenedione, testosterone, dihydrotestosterone (DHT), and estrone (E1), as measured using sensitive and specific gas chromatography-tandem mass spectrometry (GC-MS/MS), were higher in women with PCOS than in controls (Table 1 and Fig. 1B,C). We also analyzed adipose tissue concentrations of the same panel of sex steroids as in the circulation. Adipose tissue concentrations of androstenedione and testosterone were significantly higher, and there was a tendency of higher E1 levels in women with PCOS than in controls (Table 1 and Fig. 1D,E). As previously described, a single bout of 45 minutes of low-frequency electroacupuncture increased the whole body glucose uptake, measured by the euglycemic hyperinsulinemic clamp technique, in women with PCOS and in controls^18^.

**Table 1:** Circulating and adipose tissue concentrations of sex steroids measured by GC-MS/MS in women with PCOS and controls pair-wise matched for age, body weight, and body mass index.

Variable	Controls (*n* = 21)	PCOS (*n* = 21)	P*
Age (years)	29.76 ± 6.36	31.19 ± 5.56	0.520
Weight (kg)	84.64 ± 11.35	85.11 ± 13.60	0.902
BMI (kg/m^2^)	30.41 ± 3.62	31.2 ± 4.12	0.534
Triglycerides (mmol/l)	0.85 ± 0.31	1.20 ± 0.52	**0.012**
**Circulating sex steroids**			
DHEA (pg/ml)	5599 ± 4111	6873 ± 3375	0.283
DHT (pg/ml)	75.89 ± 29.2	100.7 ± 32.3	**0.016**
E1 (pg/ml)	46.93 ± 16.54	69.48 ± 33.58	**0.008**
E2 (pg/ml)	57.35 ± 41.76	87.12 ± 63.16	0.080
Progesterone (pg/ml)	822 ± 2622	1186 ± 2628	0.672
**Adipose tissue sex steroids**			
DHEA (pg/g)	20238 ± 9 606	22381 ± 521	0.538
DHT (pg/g)	3461 ± 9434	9500 ± 28110	0.232
E1 (pg/g)	350.0 ± 157.8	521.6 ± 273.2	0.058
E2 (pg/g)	209.9 ± 333.2	161.7 ± 115.8	0.285
Progesterone (pg/g)	8327 ± 16809	10226 ± 22790	0.753

P* Fisher permutation test. DHEA, dehydroepiandrosterone; DHT, dihydrotestosterone; E1, estrone; E2, estradiol.

### Changes in adipose tissue gene expression after a single bout of electroacupuncture

**Figure 2:**
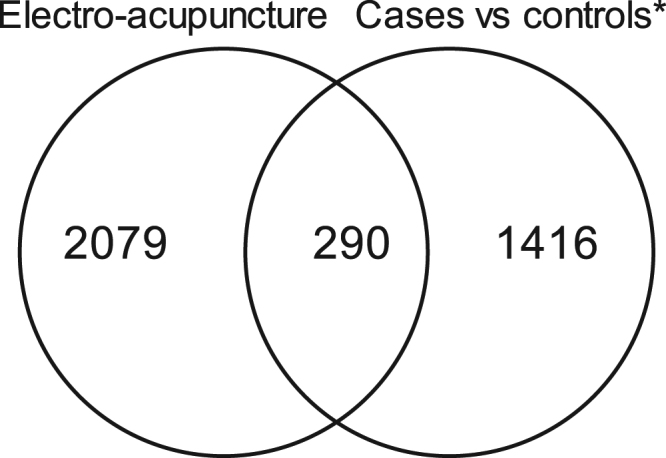
Venn diagram illustrating the overlap in significant changes in gene expression caused by a single bout of electroacupuncture and significantly differently expressed genes between women with and without PCOS (Baseline)^9^.

After applying false discovery rate (FDR) correction, we identified mRNA expression changes in 2369 unique genes (*Q* < 0.05) in adipose tissue taken before versus after a single bout of low-frequency electroacupuncture (Supplementary Table 1). Of the 2369 unique genes, 1288 were upregulated (9.4 to 2404.7%) and 1081 were downregulated (−7.8 to −53.7%). When comparing the genes affected by electroacupuncture with the 1706 genes previously shown to be differentially expressed between PCOS cases and controls^9^, 290 genes were regulated by electroacupuncture (Fig. 2, Supplementary Table 2). In total 80 genes showed a reversed expression; thus the mRNA levels after electroacupuncture were similar to those of non-PCOS controls (Supplementary Table 3).

**Figure 3:**
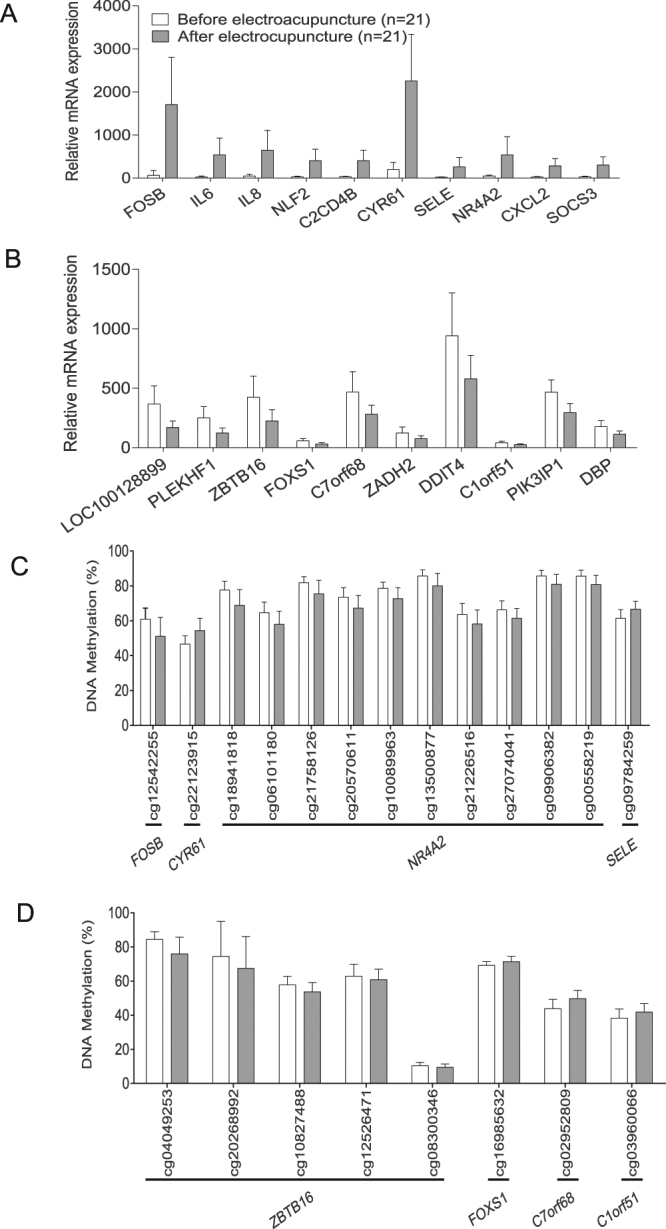
Effect of a single bout of electroacupuncture on genes with the largest changes in mRNA expression. (**A**) The largest expression changes in genes with increased mRNA levels and (**B**) The largest expression changes in genes with decreased mRNA levels relevant to PCOS. Values for gene expression are Mean ± SD. Q < 0.05. (**C**) Differential DNA methylation sites corresponding to genes with the largest increase in expression and (**D**) differential methylation sites for genes with the largest decrease in expression. Values are Mean ± SD. *Q* = 0.11.

The 25 most upregulated and 25 most downregulated genes with the largest change in adipose tissue expression after electroacupuncture are presented in Table 2. The 10 most upregulated genes (expression range 410% to 2405%) were *FOSB*, *IL6*, *IL8*, *NLF2*, *CDCD4B*, *CYR61*, *SELE*, *NR4A2*, *CXXL2* and *SOCS3* (Fig. 3A). The 10 most downregulated genes (expression range −29.8% to −53.7%) were *LOC100128899*, *PLEKHF1*, *ZBTB16*, *FOXS1*, *C7orf68*, *ZADH2*, *DDIT4*, *C1orf51*, *PIK3IP1* and *DBP* (Fig. 3B). In addition, we investigated whether top ten genes regulated by electroacupuncture had a corresponding change in methylation sites. Four methylation sites showed an inverse change whereas four changed in the same direction as the gene expression (Fig. 3C,D).

**Table 2:** Twenty-five individual genes with the largest increase and decrease in mRNA expression in adipose tissue by a single bout of electroacupuncture in twenty-one women with PCOS (*Q* < 0.05).

Gene symbol	Illumina ID	Before Single Acupuncture	After Single Acupuncture	Change (%)	*P* value	*Q* value
		Mean ± SD	Mean ± SD			
*FOSB*	1751607	68.2 ± 110.3	1709.2 ± 1094.7	2404.7	0.000	0.000
*IL6*	1699651	28.2 ± 20.7	542.5 ± 388.2	1824.3	0.000	0.000
*IL8*	2184373	51.7 ± 37.9	650.7 ± 457.8	1158.3	0.000	0.000
*NLF2*	1748751	33.7 ± 10.8	405.9 ± 265.2	1104.6	0.000	0.000
*C2CD4B*	3248551	34.5 ± 10.6	406.2 ± 242.3	1075.8	0.000	0.000
*CYR61*	2188264	202 ± 162.8	2259.3 ± 1083.5	1018.5	0.000	0.000
*SELE*	1739393	23.7 ± 3.3	263 ± 217.8	1007.5	0.000	0.000
*NR4A2*	1782305	50.5 ± 17.7	543.3 ± 416.6	976.8	0.000	0.000
*CXCL2*	1682636	27.4 ± 9.6	288.9 ± 164.1	952.4	0.000	0.000
*SOCS3*	1781001	31.6 ± 15.3	307.3 ± 185.8	873.5	0.000	0.000
*EGR1*	1762899	189.2 ± 231.7	1756.5 ± 828.5	828.3	0.000	0.000
*CCL2*	1720048	327.6 ± 423.7	2608 ± 1484.5	696.0	0.000	0.000
*ATF3*	2374865	88.3 ± 67.3	688.5 ± 344	680.0	0.000	0.000
*PTGS2*	2054297	42.6 ± 32.5	322.9 ± 225.2	657.4	0.000	0.000
*FOS*	1669523	193.5 ± 195.9	1411.2 ± 914.7	629.1	0.000	0.000
*SLC2A3*	1775708	273.3 ± 144.5	1843.7 ± 898.2	574.7	0.000	0.000
*ADAMTS1*	1673566	179.4 ± 58.3	1058.5 ± 478.8	490.0	0.000	0.000
*EGR2*	1743199	55.3 ± 27.2	313.5 ± 175.3	466.4	0.000	0.000
*MYC*	2110908	102.2 ± 48.4	543.5 ± 272.9	431.5	0.000	0.000
*JUNB*	2086077	53.3 ± 20.9	282.9 ± 150.8	430.5	0.000	0.000
*APOLD1*	1723522	216.4 ± 82.9	1147.4 ± 553.1	430.2	0.000	0.000
*CH25H*	1741021	32.3 ± 11.1	169.3 ± 99.3	424.8	0.000	0.000
*RCAN1*	1712112	48 ± 21.4	249.5 ± 177.5	419.6	0.000	0.000
*LOC387763*	1677402	318.7 ± 178.2	1645.7 ± 755.3	416.4	0.000	0.000
*EGR3*	1722781	24.2 ± 5	123.3 ± 63.4	410.0	0.000	0.000
*LOC100128899*	3269484	369 ± 150.7	170.8 ± 53.8	−53.7	0.000	0.000
*PLEKHF1*	1708041	251.7 ± 93.7	124.2 ± 41	−50.7	0.000	0.000
*ZBTB16*	2402817	426.3 ± 175.4	225.6 ± 93.8	−47.1	0.000	0.000
*FOXS1*	1811790	58.6 ± 17.7	32.4 ± 10.2	−44.8	0.000	0.000
*C7orf68*	1659990	468.8 ± 170.5	281 ± 76.9	−40.1	0.000	0.000
*ZADH2*	1795063	124.3 ± 49.8	76.6 ± 24.2	−38.4	0.000	0.001
*DDIT4*	1661599	942.8 ± 359.6	580.9 ± 196	−38.4	0.000	0.000
*C1orf51*	1793543	42.7 ± 13.3	26.6 ± 6.4	−37.7	0.000	0.000
*PIK3IP1*	1719986	468.1 ± 103.2	296.1 ± 75.3	−36.7	0.000	0.000
*DBP*	1715555	177.6 ± 50.3	113.4 ± 27.5	−36.1	0.000	0.000
*PFKFB3*	1660847	593.2 ± 279.6	382.7 ± 125.9	−35.5	0.000	0.003
*AXIN2*	1724480	361.7 ± 134.9	235.5 ± 73.9	−34.9	0.000	0.000
*SMAD7*	2203891	212.4 ± 83.4	138.5 ± 44	−34.8	0.000	0.000
*C3orf54*	1690454	119.7 ± 27.4	79.1 ± 16.8	−33.9	0.000	0.000
*FAM13A*	2401253	92.2 ± 55.9	61 ± 20	−33.8	0.000	0.000
*FLJ27365*	1815114	72.7 ± 22.6	48.2 ± 7.7	−33.7	0.000	0.000
*CLEC4GP1*	1740502	56.5 ± 17.4	37.8 ± 18	−33.2	0.000	0.002
*GIMAP7*	1776678	1084.7 ± 280.5	737.5 ± 175.4	−32.0	0.000	0.000
*TEF*	1706511	308.8 ± 57.7	210.1 ± 54.8	−32.0	0.000	0.000
*EFNA1*	2371055	733.5 ± 162.1	504.1 ± 89.9	−31.3	0.000	0.000
*PDK4*	1684982	792.3 ± 201	546.5 ± 216.5	−31.0	0.000	0.000
*PPP1R3C*	1736670	582.3 ± 336.9	404.8 ± 213.8	−30.5	0.000	0.004
*MAF*	1722206	107.7 ± 30.6	75.3 ± 29.3	−30.1	0.000	0.001
*HOXC9*	1718898	158.4 ± 31.8	110.8 ± 18.8	−30.0	0.000	0.000
*ZNF573*	1658080	144.5 ± 36	101.4 ± 26.4	−29.8	0.000	0.003

**Figure 4:**
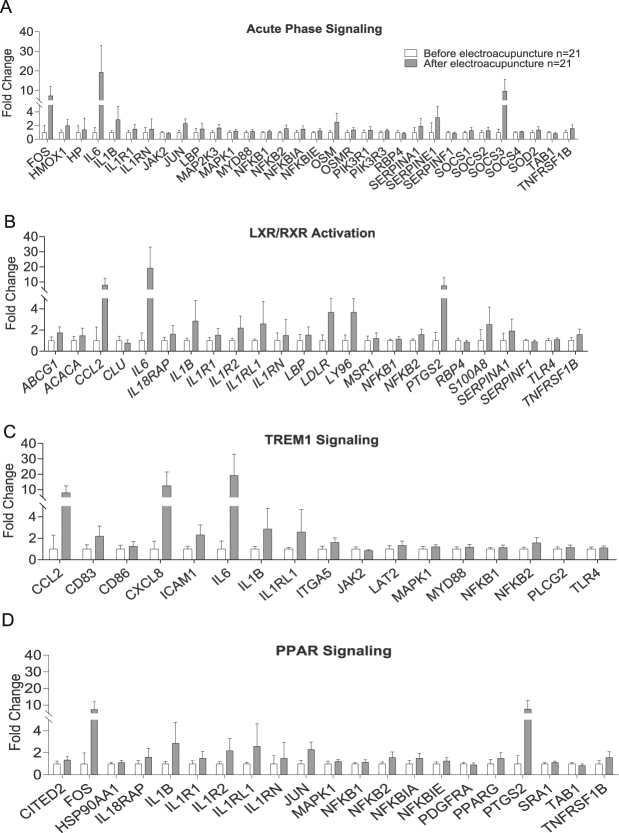
Top four gene sets activated by a single bout of electroacupuncture contributing to significant top canonical pathways, identified by Ingenuity Pathway Analysis, of possible relevance to PCOS and T2D. (**A**) Acute Phase Signaling Pathway, (**B**) TREM1 Signaling (**C**) LXR/RXR Activation Pathway, (**D**) PPAR*γ* signaling pathway. Values are presented as Fold Change. *Q* < 0.05.

Next, all genes with changed expression by electroacupuncture (*Q* < 0.05) were analyzed with IPA to identify biological pathways. All significant gene sets (*Q* < 0.05) with changed gene expression in adipose tissue taken before versus after a single bout of electroacupuncture (Table 3), and all genes contributing to the enrichment of the pathways are presented in Supplementary Tables 4 and 5. Genes contributing to the significant enrichment of the four most implicated pathways are illustrated in Fig. 4A–D.

**Table 3:** Significant gene sets with changed gene expression in adipose tissue from before to after a single bout of electroacupuncture in women with PCOS after Benjamin- Hochberg p-value correction for multiple testing. Ingenuity Pathway Analysis analyses with *Q* < 0.05.

Pathway	Regulated/Total	Ratio	Z-score	*P* value	Signaling Pathway Category	Top Functions & Diseases
*Up-regulated pathways*						
Acute Phase Response Signaling	32/103	0.311	2.043	1.36E-5	Cytokine Signaling; Ingenuity Toxicity List Pathways	Cellular Movement; Hematological System Development and Function Immune Cell Trafficking
TREM1 Signaling	17/40	0.425	3.638	1.66E-5	Cellular Immune Response; Cytokine Signaling	Cell-To-Cell Signaling and Interaction; Hematological System Development and Function; Immune Cell Trafficking
Leukocyte Extravasation Signaling	36/123	0.293	4.004	1.78E-5	Cellular Immune Response	Cellular Movement; Cell-To-Cell Signaling and Interaction; Hematological System Development and Function
IL-6 Signaling	27/85	0.318	3.53	4.08E-5	Cellular Immune Response; Cytokine Signaling	Cellular Development; Cellular Growth and Proliferation; Hematological System Development and Function
Production of NO and ROS in Macrophages	34/119	0.286	3.182	5.21E-5	Cellular Immune Response	Cell Signaling; Small Molecule Biochemistry; Free Radical Scavenging
Fcγ Receptor-mediated Phagocytosis in Macrophages and Monocytes	22/65	0.338	3.838	7.16E-5	Cellular Immune Response	Inflammatory Response; Cellular Function and Maintenance; Cell-To-Cell Signaling and Interaction
Protein Kinase A Signaling	49/205	0.239	0.493	2.14E-4	Intracellular and Second Messenger Signaling	Cellular Growth and Proliferation; Tissue Development; Tissue Morphology
NRF2-mediated Oxidative Stress Response	33/123	0.268	2.5	2.50E-4	Cellular Stress and Injury	Cell Death and Survival; Organismal Survival; Post- Translational Modification
MIF Regulation of Innate Immunity	11/25	0.44	2.714	3.67E-4	Cellular Immune Response	Lipid Metabolism; Small Molecule Biochemistry; Cell-To-Cell Signaling and Interaction
Dendritic Cell Maturation	27/96	0.281	4.315	3.96E-4	Cellular Immune Response; Cytokine Signaling; Pathogen-Influenced Signaling	Cellular Development; Hematopoiesis; Cell-To-Cell Signaling and Interaction
Toll- Like Receptor Signaling	16/47	0.34	2.714	6.32E-4	Apoptosis; Cellular Immune Response; Humoral Immune Response; Pathogen-Influenced Signaling	Infectious Diseases; Organismal Injury and Abnormalities; Renal and Urological Disease
Prolactin Signaling	18/57	0.316	0.943	8.24E-4	Cytokine Signaling; Organismal Growth and Development	Embryonic Development; Organismal Development; Cell Death and Survival
MIF-mediated Glucocorticoid regulation	9/20	0.45	2.121	1.04E-3	Cellular Immune Response; Nuclear Receptor Signaling	Hematological Disease; Infectious Diseases; Organismal Injury and Abnormalities
Role of IL-17F in Allergic Inflammatory Airway Diseases	9/21	0.429	3	1.59E-3	Cytokine Signaling; Disease-Specific Pathways	Cell-To-Cell Signaling and Interaction; Cellular Movement; Immune Cell Trafficking
B Cell Receptor Signaling	28/110	0.255	4.041	1.72E-3	Humoral Immune Response	Cellular Development; Cellular Growth and Proliferation; Hematological System Development and Function
Aryl Hydrocarbon Receptor Signaling	24/90	0.267	2	1.83E-3	Apoptosis; Cell Cycle Regulation; Ingenuity Toxicity List Pathways; Nuclear Receptor Signaling; Xenobiotic Metabolism	Cell Cycle; Gene Expression; Cell Death and Survival
HMGB1 Signaling	23/85	0.271	3.71	1.83E-3	Cellular Immune Response; Cellular Stress and Injury; Cytokine Signaling; Humoral Immune Response	Cell-To-Cell Signaling and Interaction; Cellular Movement; Hematological System Development and Function
B-cell Activating Factor Signaling	10/26	0.385	1.89	2.34E-3	Cellular Growth, Proliferation and Development; Humoral Immune Response	Cellular Development; Cellular Growth and Proliferation; Hematological System Development and Function
April Mediated Signaling	10/26	0,385	1.265	2.34E-3	Apoptosis	Cellular Development; Cellular Growth and Proliferation; Hematological System Development and Function
Tec Kinase Signaling	26/103	0.252	4.082	2.80E-3	Intracellular and Second Messenger Signaling	Cell Death and Survival; Cell-To-Cell Signaling and Interaction; Cellular Function and Maintenance
Type I Diabetes Melitus Signaling	19/68	0.279	1.698	2.99E-3	Apoptosis; Disease- Specific Pathways	Cellular Development; Cellular Growth and Proliferation; Hematological System Development and Function
Role of NFAT in Regulation of the Immune Response	27/109	0.248	2.985	3.13E-3	Cellular Immune Response; Humoral Immune Response; Intracellular and Second Messenger Signaling	Cellular Development; Cellular Growth and Proliferation; Embryonic Development
*Down-regulated pathways*						
LXR/RXR Activation	24/67	0.358	−3.838	1.51E-5	Ingenuity Toxicity List Pathways; Nuclear Receptor Signaling	Lipid Metabolism; Molecular Transport; Small Molecule Biochemistry
PPAR Signaling	21/66	0.318	−2.4	2.78E-4	Nuclear Receptor Signaling	Gene Expression; Cancer; Organismal Injury and Abnormalities

Next, we looked for overlaps between genes with changed expression by electroacupuncture (*Q* < 0.05) and genes linked to PCOS and metabolic abnormalities, including insulin resistance, type 2 diabetes, and obesity, in the genome-wide association studies (GWAS) catalog (assessed on May 29, 2017; *P* < 5 × 10^−8^; http://www.ebi.ac.uk/gwas/). Electroacupuncture changed the expression in adipose tissue in 5 out of 133 candidate genes for insulin resistance; 23 out of 470 type 2 diabetes candidate genes, and 57 out of 1205 obesity candidate genes (Supplementary Table 6). No PCOS associated candidate gene changed in adipose tissue by electroacupuncture.

**Figure 5:**
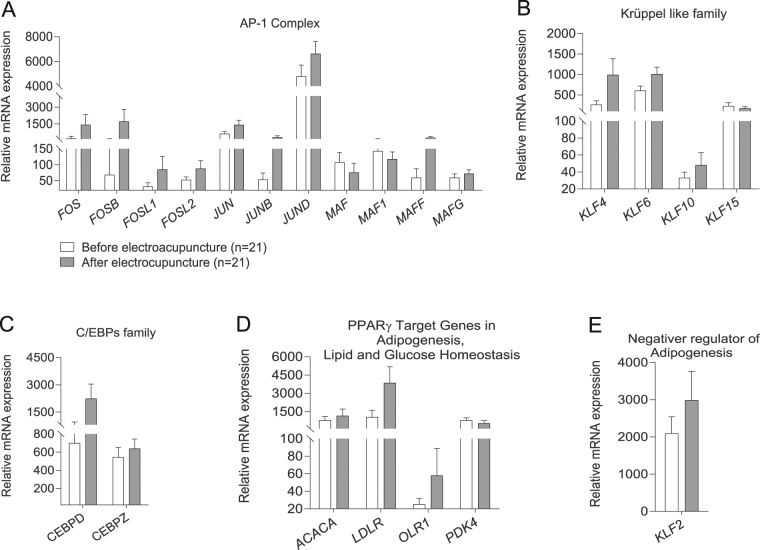
Effects of a single bout of electroacupuncture on mRNA levels of transcription factors involved in the regulation of adipogenesis: Positive regulators of adipocyte development include the transcription factor AP-1 (Activator protein-1) family (**A**); Kruppel- like family of transcription factors (**B**); C/EBP transcription family (**C**) and Transcription factors targeting PPAR- family (**D**). Negative regulators of adipose cell differentiation (**E**). Values for gene expression are Mean ± SD. *Q* < 0.05.

When applying a targeted approach to investigate if a single bout of electroacupuncture regulates positive effectors of adipogenesis, we found that electroacupuncture changed the expression of several key transcription factors essential for the recruitment of preadipocytes to differentiate into mature adipocytes in a positive direction (Fig. 5A–D). Only one transcription factor that is a negative regulator of fat cell differentiation, *KLF2*, showed altered expression after electroacupuncture (Fig. 5E).

### Changes in the expression of Nr4a2 and Junb are reversed by adrenergic blockers in rats

**Figure 6:**
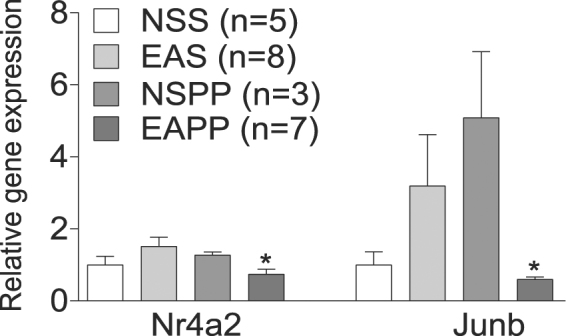
Gene expression quantification measurements of *Nr4a2* and *Junb* in inguinal fat in rats. Rats were divided into two experiments receiving electroacupuncture (EA) or no stimulation (NS) and were administrated saline (S) or a combination of nonselective α and β-adrenergic blocking agents Phentolamine and Propranolol (PP). Comparisons were made between groups receiving EASS and EAPP; *indicates *P* < 0.05 (Mann- Whitney U test). Values are Mean ± SEM.

Among the genes with differential expression after electroacupuncture (Table 2), we selected genes involved in hormonal and metabolic alterations, i.e. *IL6*, *CYR61*, *NR4A2*, *ERG1*, *CCL2*, and *JUNB*, for further mechanistic studies in rat subcutaneous adipose tissue. As α-adrenergic and β-adrenergic receptor blockade can reverse the electroacupuncture-induced increase in glucose uptake, we investigated whether the expression of the selected genes was affected by adrenergic blocking. The expression of nuclear receptor *Nr4a2* and transcriptional regulator *JunB* in inguinal adipose tended to increase by low-frequency electroacupuncture but did not reach significance (*P* = 0.127 and *P* = 0.14 respectively) (Fig. 6). Administration of combined α- and β-adrenergic blocking agents decreased the expression of *Nr4a2* and *JunB* compared to electroacupuncture (*P* < 0.05, Fig. 6). The expression of *FosB*, *Cyr61*, *Egr1*, and *Ccl2*, did not change (Fig. S1).

### Changes in genome-wide DNA methylation in adipose tissue

**Figure 7:**
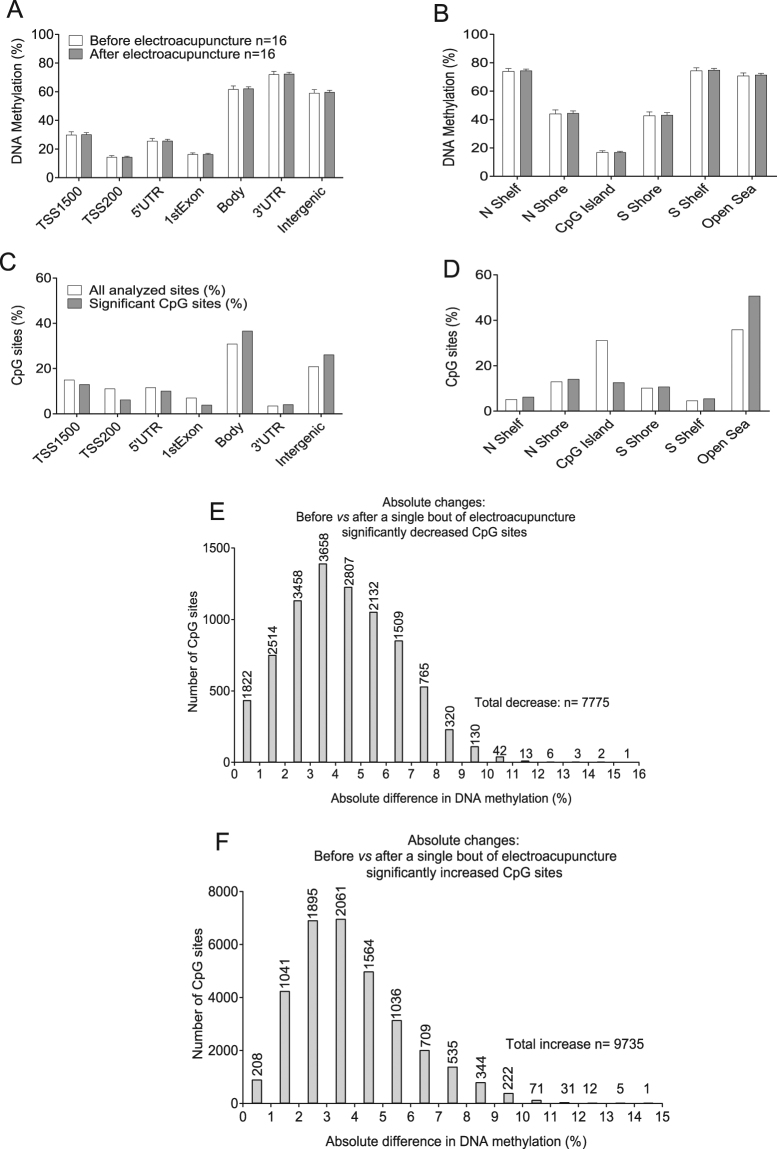
Effects of a single bout of electroacupuncture on global DNA methylation in human subcutaneous adipose tissue in women with PCOS. Global DNA methylation was calculated as the average DNA methylation of all CpG sites in each annotated region on the Infinium Human Methylation 450 BeadChip presented for (**A**) the nearest gene region and (**B**) the nearest CpG island region (mean ± SD). The distribution of significant sites compared to all analyzed sites in relation to (**C**) the nearest gene region and (**D**) the nearest CpG island region. The absolute difference in DNA methylation of 17, 510 individual CpG sites in adipose tissue from 16 women with PCOS after a single bout of electroacupuncture divided into (**E**) sites with decreased methylation and (**F**) sites with increased methylation. TSS, proximal promoter defined as 200 or 1500 bp upstream of the transcription site; Shore, flanking region of CpG island (0–2000 bp from the CpG island); Shelf, regions flanking island shores (2000–4000 bp from the CpG island).

Genome-wide DNA methylation patterns after a single bout of low-frequency electroacupuncture were analyzed in adipose tissue from 16 out of the 21 women with PCOS. We identified DNA methylation changes in 93,260 CpG sites (*P* < 0.05), which constitutes 19% of all 483,317 analyzed sites (Supplementary Table 7). This is 3.9 times higher than expected by chance (chi-square test *P* < 0.0001). However, none of the identified sites were significantly altered if applying an FDR correction based on *Q* < 0.05. Therefore, for further analyses, we focused on the 17,510 CpG sites identified as altered using an FDR cutoff of *Q* = 0.11 (no sites significance with a *Q* < 0.10) (Supplementary Table 8). This is about 30% less than expected by chance (chi-square test *P* < 0.0001). Due to the potential problems with Infinium probes, we calculated potential cross-reactive probes in our methylation data set (Supplementary Table 9) according to a previous report^19^. To evaluate effects of location in the global methylome, we calculated the average level of methylation for all sites divided into groups based on either their location in relation to the nearest gene (Fig. 7A) or their location in relation to CpG islands (Fig. 7B). There were no significant differences in average DNA methylation for the different types of regions when comparing before versus after a single bout of electroacupuncture.

Differentially methylated sites were over-represented in the gene body, intergenic regions, and open sea, and under-represented in CpG islands compared to the probe distribution on the array (Fig. 7C,D). The absolute change in DNA methylation, calculated from before to after electroacupuncture, showed decreased methylation in 7775 sites, ranging from −0.2 to −15% (Fig. 7E) and increased methylation in 9735 sites, range 0.2 to 14% (Fig. 7F).

Next, we overlapped the identified DNA methylation sites changed by electroacupuncture (*Q* < 0.11) with genes linked to PCOS and metabolic abnormalities, including insulin resistance, type 2 diabetes, and obesity, in the genome-wide association studies (GWAS) catalog (assessed on May 29, 2017; *P* < 5 × 10^−8^; http://www.ebi.ac.uk/gwas/). We found that electroacupuncture altered the DNA methylation in 36 out of 133 candidate genes for insulin resistance; in 110 out of 470 type 2 diabetes candidate genes, in 281 out of 1205 obesity candidate genes, and in 7 out of 21 PCOS candidate genes (Supplementary Table 10).

### Overlap and correlations between gene expression and DNA methylation

**Figure 8:**
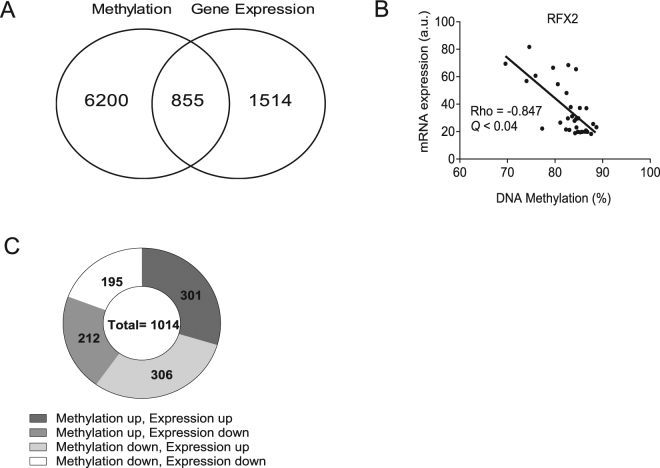
Venn diagram illustrating (**A**) Overlap between changes in gene expression and DNA methylation caused by a single bout of electroacupuncture. (**B**) Overlapping data of significant changes according to the direction of the changes in methylation and gene expression induced by a single bout of electroacupuncture. (**C**) Correlations of gene expression with DNA methylation in adipose tissue after a single bout of electroacupuncture. Expression of regulatory factor X variant 2 (*RFX2) gene:* a transcription factor, correlates negatively with DNA methylation (cg04512965).

In a combined analysis of the DNA methylation (*Q* = 0.11) and gene expression (*Q* < 0.05) data (Fig. 8A), we found overlaps in 10% of common genes between these two data sets. Out of the 855 significant gene overlaps (corresponding to 964 sites), we found a decreased mRNA expression at 398 sites and an increase at 566 sites (Supplementary Table 11). Next, all 855 individual genes were sorted according to the direction of the changes. Out of the 1014 significant overlaps between genes with changed expression after electroacupuncture and their annotated DNA methylation sites, approximately half of all gene expression changes were accompanied by an opposite change in DNA methylation (Fig. 8B). DNA methylation is usually associated with gene repression, and here we found that hypermethylation in 306 single CpG sites was associated to decreased gene expression. However, we also found an increased gene expression in 301 individual hypermethylated sites, which suggest that splicing or alternative promoters regulate the expression of these genes^20^.

Next, we performed Spearman correlation analyses (Supplementary Table 14), with correction for multiple testing, to investigate correlations between the 2369 significant differential changes in gene expression (*Q* < 0.05) and differential changes in DNA methylation (*Q* < 0.11). Out of 407 significant correlations (*P* < 0.05) 205 genes showed negative association (*Rho* = −0.05 to −0.85) between gene expression and DNA methylation and 202 genes displayed positive correlation (Rho = 0.05– to 0.83) where an increase in methylation is associated with an increase in expression. After FDR corrections, only one gene, the transcription regulatory Factor X (*RFX2*), showed a significantly negative correlation between expression (ILMN_2396287) and methylation (cg04512965); (*Rho* = −0.85; Q < 0.04; Fig. 8C).

### Correlations between changes in the glucose infusion rate, gene expression, and DNA methylation

To investigate if the electroacupuncture-induced changes in the glucose infusion rate (GIR), as measured by a euglycemic hyperinsulinemic clamp, are correlated with changes in gene expression and/or DNA methylation, we performed correlation analyses with the 25 genes with the largest increase and the 25 with the largest decrease in gene expression. Surprisingly, only the genes with decreased expression correlated with changes in GIR and showed significant correlations between mRNA expression and their corresponding methylation sites (Table 4). Decreased mRNA expression of *ZBTB16* (r_s_ = 0.499), *PFKFB3* (r_s_ = 0.483), and *SMAD7* (r_s_ = 0.456) correlated positively with increased GIR and decreased mRNA expression of *FOXS1* (r_s_ = −0.560), *CLEC4GP1* (r_s_ = −0.570), *GIMAP7* (r_s_ = −0.496), and *MAF* (r_s_ = −0.561) correlated negatively with GIR. Increased GIR correlated positively with changes in DNA methylation of *CXCL2* (r_s_ = 0.5), *EGR3* (r_s_ = 0.544), *FOSB* (rs = 0.506), *NR4A2* (r_s_ = 0.600), *PFKFB3* (r_s_ = 0.518), and *ZBTB16* (r_s_ = 0.612) (Table 4). Furthermore, since the top 50 genes with the largest changes in gene expression are not the same as the top genes with the largest changes in DNA methylation we selected the top 25 hyper- and top 25 hypomethylated genes and correlated with changes in GIR. Significant correlations are presented in Supplementary Tables 12 and 13.

**Table 4:** Correlations between changes in the glucose infusion rate and the 25 genes with the largest changes in expression (n = 21; *Q* < 0.05) and their corresponding methylation sites (n = 16; *Q* = 11) after a single bout of electroacupuncture in women with PCOS.

Gene symbol	Illumina ID	Correlation Coefficient	Expression Significance	Target ID	Gene Region	Correlation Coefficient	Methylation Significance
*CLEC4GP1*	1740502	−0.570	0.007	cg24889914	TSS200	−0.703	0.002
				cg16715857	Body	−0.524	0.037
*FOXS1*	1811790	−0.560	0.008			ns	ns
*GIMAP7*	2133316	−0.496	0.022			ns	ns
*MAF*	1722206	−0.561	0.008			ns	ns
*PFKFB3*	1660847	0.483	0.027	cg16179674	Body	0.518	0.04
*SMAD7*	2203891	0.456	0.038			ns	ns
*ZBTB16*	2402817	0.444	0.044	cg20268992	Body	0.612	0.012
*ZBTB16*	2305407	0.499	0.021	cg04049253	Body	0.568	0.022
*APOLD1*		ns	ns	cg16747828	Body; TSS200	−0.703	0.002
*C7orf68*		ns	ns	cg02952809	TSS1500	−0.691	0.003
*CCL2*		ns	ns	cg04633676	TSS1500	−0.597	0.015
*CXCL2*		ns	ns	cg18356190	Body	0.500	0.049
*EGR3*		ns	ns	cg08810842	Body	0.521	0.039
*EGR3*		ns	ns	cg07082452	3′UTR	0.538	0.031
*EGR3*		ns	ns	cg03127416	Body	0.521	0.039
*EGR3*		ns	ns	cg14394550	Body	0.544	0.029
*FOS*		ns	ns	cg23404711	Body	−0.521	0.039
*FOSB*		ns	ns	cg12542255	Body	0.506	0.046
*NR4A2*		ns	ns	cg18941818	Body	0.500	0.049
*NR4A2*		ns	ns	cg06101180	Body	0.509	0.044
*NR4A2*		ns	ns	cg21758126	Body	0.600	0.014
*NR4A2*		ns	ns	cg20570611	Body	0.506	0.046
*SELE*		ns	ns	cg09784259	TSS1500	−0.738	0.001

## Discussion

This study shows the effect of a single bout of electroacupuncture on genome-wide DNA methylation and gene expression in subcutaneous adipose tissue in women with PCOS. This is the first study to demonstrate that circulating androgen concentration is directly reflected by the adipose tissue androgen content in age, weight, and BMI matched cases and controls. Based on microarray data, we provide the first evidence that a single bout of low-frequency electroacupuncture treatment, mimicking muscle contraction, induces global changes in adipose tissue DNA methylation and gene expression. We have previously demonstrated that women with PCOS have alterations in their adipose tissue DNA methylation patterns by up to 6.3%, and we proposed that those alterations could be involved in changing gene expression and explain the metabolic phenotype in women with PCOS^9^. Here we tested the hypothesis that a single bout of low-frequency electroacupuncture has the capacity to restore methylation and gene expression disturbances, which could improve whole body glucose homeostasis in a similar way as exercise by inducing muscle contractions. It is largely accepted that aberrant DNA methylation is involved in the development of metabolic diseases, such as type 2 diabetes^21^ and obesity^22^, and that physical exercise can improve a large number of different health related outcomes^23^. Furthermore, DNA methylation is a dynamic process, and aberrant DNA methylation patterns can be modulated by exercise^13,24^.

In this study, methylation changes ranged from 1% up to 16%, which corresponds to approximately 3.5% of all analyzed CpG sites and expression alterations were up to 2404.7%. When we merged the methylation and gene expression results, we found overlaps for 855 individual genes. Furthermore, when we analyzed according to the direction of the changes, we observe that electroacupuncture drives the DNA methylation to increase in 513 sites while it was decreased in 501 sites. These key findings demonstrate that electroacupuncture has an acute and global effect on both the methylome and the transcriptome. We also found 407 significant correlations between DNA methylation (*Q* < 0.11) and gene expression (*P* < 0.05), although only one gene, the *RFX2*, remained significant after FDR corrections. Electroacupuncture decreased the methylation in the body region of the gene by 4.24% and increased the expression of *RFX2* by approximately 91.8%. This gene is involved in the modification of cellular motility (spermiogenesis)^25^. First-degree male relatives of women with PCOS often have metabolic disturbances^26^. However, if there is a connection between PCOS and the spermiogenesis of sons to women with PCOS needs to be investigated. Although it is unknown if there is a link between adipose tissue expression of genes involved in spermiogenesis and sperm function, acupuncture has been shown to improve sperm function in infertile patients^27^.

The large changes in gene expression observed are most likely due to multiple epigenetic mechanisms such as histone post-translational modification and non-coding RNAs, in addition to the changes in DNA methylation. Since a single bout of electroacupuncture causes an increased whole-body glucose uptake, we explored if this was related to changes in methylation. Interestingly, DNA methylation changes within the body region of several genes correlated with changes in glucose uptake, whereas only a small fraction of genes with methylation changes in the regulatory regions correlated with changes in glucose uptake.

We found that the hypomethylated gene lipin1 (*LPIN1*) correlated negatively with GIR and that the regulatory associated protein mTOR complex 1 (*RPTOR*) correlated positively with GIR in response to one single bout of electroacupuncture. The effect of acute exercise on methylation levels in these genes in adipose tissue has not previously been described. However, it was found that the expression profile of LPIN1 and RPTOR in the liver is improved after 12 weeks of exercise in an obese mouse model^28^. A Lipin1 polymorphism has been associated with PCOS suggesting its involvement in metabolic complications^29^.

Among the hypermethylated genes, we found that two methylation sites for dual specificity phosphatase 1 (*DUSP1*) correlated significantly with GIR. Of interest, animal and human studies have demonstrated increased mRNA levels of *DUSP1* in subcutaneous adipose tissue contributing to decreased fatty acid oxidation and decreased protection against high fat diet-induced inflammation^30,31^. In addition, three months of physical exercise reduced the expression of *DUSP1*, with a parallel increase in the expression of *PGC1α*, and improved overall metabolic homeostasis^32^. The ZFP36 ring finger protein (*ZFP36*) showed changes in gene expression after repeated exercise. This is in line with what we observed in the present study, where *ZFP36* methylation changes correlated negatively with GIR. Furthermore, genes with the largest decrease in gene expression correlated with changes in whole-body glucose uptake, however, no correlations were found among genes with increased expression.

Of note, the genes with decreased expression after electroacupuncture are mainly expressed in stromal cells. The metabolic role of the connective tissue surrounding adipocytes is still not well established, but its role in extracellular matrix remodelling during adipose tissue expansion is under investigation. The genes with increased expression after one bout of electoacupuncture are mostly expressed in inflammatory cells such as T and B cells and none of these genes correlated with insulin stimulated glucose uptake. It has been suggested that inflammation, angiogenesis, and the extracellular matrix contribute to both “healthy” and “unhealthy” adipose tissue expansion^33^, and electroacupuncture may affect these processes rather than GIR in adipose tissue. These findings suggest that changes in DNA methylation might play a role in the regulation of glucose uptake. Further, functional *in vitro* studies demonstrate that *HDAC4* regulates glucose uptake in 3T3-L1-cells^14^, and in line with that observation, *HDAC4* gene expression and DNA methylation was regulated by electroacupuncture in the present study.

To investigate if electroacupuncture had an impact on restoring the aberrant gene expression in women with PCOS, we looked for overlaps between genes affected by electroacupuncture and genes previously reported as differently expressed between cases and controls^9^. In total 80 genes were reverted to more healthy phenotype by electroacupuncture, including *PPARγ*, *ADIPOR2*, and *CD74*^9^. *PPARγ* is a critical player in the regulation of glucose metabolism, lipid storage, inflammation, cancer, and the regulation of whole body energy homeostasis^34^. It is crucial for adipocyte development *in vitro* and *in vivo*^35^. In our previous case-control study of PCOS^9^, we found association with an increase in methylation in different regions of the *PPARγ* gene. This increase in methylation was associated with a decrease in the gene expression by 13.5% which supports our hypothesis that women with PCOS have epigenetic dysfunctions in *PPARγ* that, at least in part, contribute to the development of insulin resistance^9^. Interestingly, electroacupuncture decreased methylation marks in the body regions of *PPARγ* by 5.7% and increased the expression by 49.7%, which indicates that electroacupuncture exerts similar changes on *PPARγ* mRNA levels as exercise and in part explains the increased glucose uptake measured by the clamp^36,37^.

Furthermore, there is evidence that disruption of adiponectin and adiponectin signaling can contribute to the pathogenesis of PCOS^38^. Adipocyte size together with circulating adiponectin are the strongest factors explaining insulin resistance in women with PCOS^39^. A previous study reported that expression of adiponectin receptor 2 in women with PCOS is downregulated by hyperinsulinemia and overweight^40,41^. Here we demonstrate that electroacupuncture increases the *ADIPOR2* mRNA expressions by 21% and therefore might be considered as an alternative treatment to improve glucose and lipid uptake *via* increased adiponectin signaling.

Major histocompatibility complex, class II invariant chain (*CD74*) is involved in the regulation of adipogenesis and inflammation^42^, and we have demonstrated increased expression of *CD74* in adipose tissue from women with PCOS, which might contribute to the unhealthy PCOS phenotype^9^. In this study, the *CD74* gene was hypomethylated by 2.4%, and mRNA levels were decreased by ~23%, supporting the idea that electroacupuncture may have the capacity to restore methylation alterations and lead to improved metabolic health in women with PCOS.

Next, we investigate the impact of electroacupuncture on genes previously reported as altered in adipose tissue from women with PCOS^43^. Electroacupuncture increases the expression of *FOSB* by 2404.7%, jun B proto-oncogene (*JUNB)* by 430.5% and C motif chemokine ligand (*CCL2)* by 696% in a similar way as exercise^44,45^. Interleukin-6 (*IL6)* expression increased by 1824.3%, which might be the result of muscle contractions in combination with muscle and adipose tissue cross–talk, leading to direct effects on both lipolysis and fatty acid oxidation^46^. The increase in *IL6* expression could be a result of hyperinsulinemia during the euglycemic hyperinsulinemic clamp^47^. However, as biopsies were collected under the same hyperinsulinemic conditions, it is not expected that the clamp *per see* affects gene expression, but it cannot be entirely ruled out. The significance and importance of induction of the pro- and anti-inflammatory chemokines by both exercise and electroacupuncture needs further evaluation^48^. The decrease in the methylation of the nuclear receptor subfamily 4 group 2 (*NR4A2*) gene was associated with a 976.8% increase in mRNA expression. The biological function of NR4A receptors in adipose tissue and adipocytes remain unclear, but interestingly, the NR4A receptor family has been associated with glucose utilization and oxidative phosphorylation in liver and skeletal muscle^49,50^.

We have established that a single bout of electroacupuncture increases whole body glucose uptake in overweight and obese women with and without PCOS, an effect that at least in part is mediated via activation of autonomic nervous system^18^. Therefore, we investigated whether the expression of selected genes that changed by electroacupuncture was blocked when administrating alpha- and beta-adrenergic blockers to rats during a euglycemic hyperinsulinemic clamp. Out of seven genes, the expression was blocked in *Nr4a2* and *Junb*, indicating that some of the expression changes are mediated via activation of the sympathetic nervous system.

Network analysis revealed a number of activated pathways that could be associated to acupuncture. Genes enriched in acute phase signaling and the TREM1 signaling pathway were all upregulated. Upregulation of the acute phase genes in response to one single bout of electroacupuncture is expected since activation of the pathway is a response to tissue injury. The driver of this pathway is IL-6 which is produced and might have beneficial role in skeletal muscle tissue repair and organ crosstalk^51^. Here we found that *IL6* was upregulated in adipose tissue. The metabolic role of IL-6 is extensively studied and there is no consensus if IL-6 is a pro-inflammatory marker directly involved in insulin resistance or an anti-inflammatory marker that increases insulin secretion and beta-oxidation, and improves beta cell function^52^. An emerging concept is that IL-6 appears to have different effects on different tissues and whether the levels are acutely or chronically elevated. One acute bout of electroacupuncture, causing muscle contractions, might increase mRNA levels of *IL6* in overweight women in the same way as acute exercise^53^. IL-6 either released from skeletal muscle or adipose tissue induce GLP-1 release, leading to insulin secretion, improved beta cell function and whole body glucose homeostasis^52^.

Upregulation of the genes in the TREM1 Signaling pathway in adipose tissue in response to exercise or acupuncture has not previously been demonstrated. The activation of genes involved in the regulation of PPAR and LXR/RXR signaling may have clinical implication as PPARs together with genes in RXR signaling form complexes that regulate overall metabolism and inflammation^54^.

Using a targeted approach, we discovered that electroacupuncture activates a large number of transcription factors known to be positive regulators of adipogenesis. Whether adipose tissue differentiation is disturbed in women with PCOS is unknown. Adipose tissue from prenatal androgen exposed female rhesus monkeys display dysfunctional expression of genes involved in adipocyte maturation that subsequently promotes insulin resistance through lipotoxicity^55,56^. Activated genes in the early stage of adipogenesis belong to the AP-1 complex family, *KLF4*, and *KLF6*, and two genes in C/EBP family. Furthermore, electroacupuncture increased mRNA expression of genes involved in the later stage of adipocyte differentiation by activating other factors regulating PPARγ expression.

A strength of the present study is that the included women with PCOS are well characterized and have elevated circulating androgens and enlarged adipocytes. In addition, we demonstrate, for the first time, that androstenedione and testosterone concentrations in subcutaneous adipose tissue, as measured by the highly sensitive and specific GC-MS/MS method, is higher in women with PCOS than in controls and mirrors the circulating levels. The limitation is that adipose tissue is heterogeneous and we used whole tissue for methylation and gene transcription profiling. Therefore, the results may also reflect the activity of other cells in adipose tissue, including connective tissue and immune cells. Furthermore, we used the 450k BeadChip array to investigate changes in methylated CpG sites, which interrogates only 2% of all methylation sites in the genome. Thus, it does not give us a complete understanding of the impact of acupuncture on the whole methylome and transcriptome.

In conclusion, low-frequency electroacupuncture with muscle contractions remodels epigenetic and transcriptional changes in adipose tissue that elicit metabolic improvement, an effect that in part is mediated via activation of the autonomic nervous system.

## Materials and Methods

### Study Design

The study was conducted at the Sahlgrenska Academy, University of Gothenburg, Sweden, in accordance with the Declaration of Helsinki. It was approved by the Regional Ethical Review Board of the University of Gothenburg (Dnr: 520-11). Potential participants were recruited by advertisement in a local newspaper and in public areas between January 2012 and November 2013. All participants were informed about the study design by oral and written information. After taking part of this information, all participants gave oral and written consent before inclusion. The study was registered October 6th, 2011, at ClinicalTrials.gov (NCT01457209) and is reported according to the CONSORT and STRICTA guidelines^57,58^.

### Study Population

The PCOS cohort consisted of 21 overweight – obese women (BMI >25 to <35), 18 to 38 years old, and fulfilling at least two out of the three PCOS diagnostic criteria^59^; ultrasound-verified polycystic ovaries (≥12 follicles of 2–9 mm and/or ovarian volume ≥10 mL in one or both ovaries), oligo/amenorrhea (>35 days cycles), and/or clinical signs of hyperandrogenemia (hirsutism or acne) defined by a self-reported Ferriman-Gallwey (FG) score ≥8. Twenty-one age, weight, and BMI matched controls were included to investigate differences in circulating and adipose tissue sex steroid concentrations and adipocyte size^18^. Potential participants were excluded if they had taken any pharmacological treatments in the previous 3 months, had breastfed during the 6 months prior to inclusion in the study, had received acupuncture during the last 2 months, or had a history of daily smoking or alcohol consumption. Other exclusion criteria were cardiovascular disease, diabetes mellitus, and other endocrine disorders such as congenital adrenal hyperplasia, Cushing’s syndrome, or androgen–secreting tumors.

### Study Procedure

For a Western-medicine style of acupuncture we used a fixed protocol^60^. A single bout of acupuncture with manual and low-frequency electrical stimulation of the needles causing muscle contractions, so called electroacupuncture, was given during a euglycemic-hyperinsulinemic clamp, a method that has been described in detail^18^. The procedure was performed at menstrual cycle day 1–10. If no bleeding had occurred during the period between screening and the experimental study day, measurements were taken on any arbitrary day of the cycle. In brief, after an overnight fast, basal blood samples were collected before insulin was infused (40 mU/min/kg) for 120 min to reach steady state. Blood glucose levels were measured and adjusted every fifth minute using OneTouch Ultra2 technology (LifeScan, Inc., Milpitas, CA, USA). When the steady state was reached, and before the start of the electroacupuncture, a needle biopsy of subcutaneous adipose tissue was obtained under local anesthesia (Xylocaine, AstraZeneca AB, Södertälje, Sweden). The biopsy was rinsed with saline, and one part was snap frozen in liquid nitrogen, and one part was immediately isolated to determine adipocyte size, as previously described^61,62^. At steady state, and immediately after the adipose tissue biopsy, acupuncture needles, 40 mm × 0.30 mm (HEGU Svenska AB, Landsbro, Sweden), were inserted to a depth of 15–40 mm bilaterally in abdominal muscles (Conception Vessel [CV] 3 and 12, Stomach ([ST] 29), located in the same somatic innervation area as ovaries and pancreas, and in quadriceps muscles (ST 32 and 34), with the aim to activate large muscles. In addition, needles were placed in muscles below the knee (ST36) and Spleen (SP) 6 and in the hand (Large Intestine 4). When inserted, all needles were stimulated by manual rotation until needle sensation (*de qi*). Needles in the quadriceps and abdominal muscles were attached to electrodes and electrically stimulated (CEFAR ACUS4; Cefar- Complex Scandinavia, Sweden) with low–frequency (2 Hz), causing muscle contractions. Needles not connected to the electrical stimulator were stimulated every 10 min during 45 min. Immediately after 45 minutes of electroacupuncture, a second needle biopsy of subcutaneous adipose tissue was obtained.

### Biochemical Analyses

Circulating and subcutaneous adipose tissue levels of dehydroepiandrosterone (DHEA), androstenedione, testosterone, dihydrotestosterone (DHT), estrone (E1), estradiol (E2), and progesterone were measured by GC-MS/MS as previously described^63,64^.

### Description of Animal Study

The animal experiments were approved by the Animal Ethic Committee at the University of Gothenburg, Sweden, and followed the Guide for the Care and use of Laboratory Animals. Female Wistar rats (Charles River, Frankfurt, Germany) arrived at 13 weeks of age and were fed *ad libitum* with standard chow diet (Harlan Teklad Global Diet, Frankfurt, Germany). Vaginal smear was performed to determine estrus cycle stage by microscopic analysis^65^. Rats in estrus phase were selected and subjected to a euglycemic-hyperinsulinemic clamp and randomly divided into a no-stimulation group (NS) or a low-frequency electroacupuncture group (EA). The euglycemic-hyperinsulinemic clamp was performed in anesthetized rats (150 mg/kg, i.p.; Inactin, Sigma-Aldrich, St. Louis, USA). Catheters were inserted into the right jugular vein for constant infusion of insulin (8 mU∙kg^−1^∙min^−1^) and 20% saline glucose solution to maintain a glucose concentration of 6 mmol/L. Blood was drawn from the left carotid artery, and blood glucose levels were measured every 5 minutes (OneTouch Ultra 2, LifeScan, Inc., Milpitas, USA). At steady state, two acupuncture needles (0.20 × 15 mm HEGU Svenska AB, Landsbro, Sweden) were inserted bilaterally in rectus abdominis muscle (corresponding to acupuncture points ST27 to 29) and two in each triceps surae muscles (corresponding to SP6 and SP9). All acupuncture points were in somatic segments corresponding to the same innervation area as ovaries and pancreas. After insertion, the needles were attached to an electrical stimulator (CEFAR ACU II; Cefar-Complex Scandinavia, Sweden) and were subjected to low-frequency stimulation (2 Hz), causing visible muscle contractions for 45 minutes. Responders (>15% increase in glucose infusion rate (GIR)) were identified after 25 minutes of stimulation and were further subdivided into groups that received a combination of non-selective α adrenergic (phentolamine hydrochloride, Sigma-Aldrich, Munich, Germany) and β adrenergic (propranolol, Sigma-Aldrich, Munich, Germany) blocking agents or saline. After 45 minutes of electroacupuncture, rats were followed for 60 minutes. Animals in the no stimulation group underwent the same procedure. Rats were euthanized by decapitation, and inguinal adipose tissue was harvested for molecular analyses.

### mRNA and DNA Extraction from Human and Rat Subcutaneous Adipose Tissue

From human adipose tissue, total mRNA for gene expression was extracted by the RNeasy Lipid Tissue Mini Kit (Qiagen, Hilden, Germany) and for methylation studies, DNA was extracted using the QIAamp DNA Mini Kit (Qiagen, Hilden, Germany). The concentration of mRNA was determined with a NanoDrop spectrophotometer (Thermo Scientific, Wilmington, DE). The mRNA integrity was analyzed by an Experion Automated Electrophoresis system (Bio-Rad Laboratories, Hercules, CA, USA). From rat inguinal (subcutaneous) adipose tissue, total RNA was isolated by the RNeasy Lipid Tissue Mini Kit (Qiagen, Hilden, Germany) and cDNA was prepared using the High Capacity RNA to DNA kit (Applied Biosystems, Hercules, CA, USA).

### Expression Arrays

To assess the global mRNA expression changes in human subcutaneous adipose tissue, we analyzed isolated mRNA with microarray HumanHT–12 v4 Expression BeadChip (Illumina, San Diego, CA, USA). In brief, cRNA synthesis, including biotin labeling, was carried out using the Illumina^®^ TotalPrep™ RNA Amplification Kit (Life Technologies & Invitrogen, Carlsbad, CA, USA) according to the manufacturer´s recommendations. Biotin–cRNA complexes were then fragmented and hybridized to the probes on the Illumina BeadChip array. Probes were hybridized and stained with streptavidin-Cy3 before visualization with an Illumina HiScan fluorescence camera. Paired samples were included on the same chip to avoid a batch effect. The bioinformatic analyses were performed at the SciLife Lab, Department of Medical Biochemistry and Microbiology, Uppsala, Sweden. Data were background corrected and quantile normalized using the Lumi package^66^. The Oligo package from Bioconductor was used to compute Robust Multichip average expression measures^67^. Probes were filtered based on mean detection p–value ≤0.05.

### Quantitative Real–Time PCR

SYBR Green real-time PCR reactions were performed for detection and quantification of mRNA in rats. Specific pairs of PCR primers for the following genes were used: *Fosb*, *Il6*, *Cyr61*, *Nr4a2*, *Egr1*, *Ccl2*, and *Junb* (Supplementary Table 14, Bio–Rad, Hercules, CA, USA). All reactions were performed in triplicates, 1 ng of cDNA was used in all reactions except for *Il6* (2 ng of cDNA) in combination with the Power SYBR^®^ Green PCR Master Mix (Applied Biosystem, Hercules, CA, USA) and amplified by the Applied Biosystem StepOnePlus Real-Time PCR Systems according to the manufacturer’s instructions. The NormFinder algorithm was used to calculate the most stable reference gene. Gene expression results were normalized against the expression of *Hprt1*, the endogenous control that showed the lowest variability in the inguinal fat of rat.

### DNA Methylation Arrays

Genome-wide DNA methylation in subcutaneous adipose tissue in women with PCOS was analyzed with Illumina Infinium HumanMethylation450k array BeadChips (Illumina, San Diego,CA, USA). The array contains 485,577 cytosine probes covering 21,231 (99%) RefSeq genes^68^. A Zymo Methylation Kit (D5001-D5002, Zymo Research, Irvine, CA, USA) was used to convert genomic DNA to the bisulfite-modified DNA. Briefly, gDNA (500 ng) of high quality was fragmented and hybridized on the BeadChip, and the intensities of the signals were measured with a HiScanQ scanner (Illumina, San Diego, CA, USA). The methylation values for each CpG site are presented as a β-value ranging from 0 (unmethylated) to 100% (completely methylated).

The bioinformatics analyses were performed as described^21,69^ at the Department of Clinical Sciences at Lund University in Malmö. In brief, Y chromosome probes, rs–probe, and probes with average detection *P*–value >0.01 were removed. After quality control and filtering, methylation data were obtained for 483,317 CpG sites. Beta-values were converted to M–values (M = log2 (β/ (1 − β), which were used for all data analyses. Data were then quantile normalized and batch corrected with COMBAT^70^. To improve the interpretation, after all the preprocessing steps, data were reconverted to beta-values, which are presented in tables and figures.

### Ingenuity Pathway Analysis (IPA)

Network analysis in gene expression datasets was performed using the Ingenuity Pathway Analysis tool (IPA, Redwood City, CA, USA) for prediction of upregulated and downregulated canonical pathways. A list with Illumina’s RefSeq annotation and fold change, *Q*– and *P*–values were calculated before data entry, and thereafter uploaded in IPA. *Q* < 0.05 was used as the cut–off for gene expression. As a predictor of activation and measure of significance, we used the computed z-score generated by the IPA software. A *z*–score larger than 2 is an indicator of a canonical pathway being up-regulated and, z–score less than 2 indicates down-regulation of a pathway. Furthermore, false discovery rate (Benjamin–Hochberg Multiple testing scores) was applied to the network analysis to reduce the number of false positive.

### Statistics

Changes in human adipose tissue DNA methylation and gene expression between before and after a single bout of electroacupuncture were based on linear regression analyses. False discovery rate (FDR) was used to correct for multiple testing. The chi–square test was used to calculate whether the changed methylated sites were more than the expected number by chance. The association between gene expression and DNA methylation was determined using the Spearman correlation coefficient followed by FDR corrections. Data for DNA methylation and gene expression are presented as mean ± SD. The Benjamin–Hochberg method was also used to correct for multiple testing of *P*–value in the IPA. The Mann-Whitney U test was used to determine changes in gene expression in inguinal adipose tissue in rats and data are presented as mean ± SEM. Statistical analyses were performed using the SPSS software (version 24; SPSS, Inc., Chicago, IL, USA).

### Data availability

The datasets generated during and/or analysed during the current study are available from the corresponding author on reasonable request.

### One Sentence Summary

Transcriptional and epigenetic remodeling by electroacupuncture.

## Electronic supplementary material

Supplementary info

Supplementary Table 1

Supplementary Table 2

Supplementary Table 3

Supplementary Table 4

Supplementary Table 5

Supplementary Table 6

Supplementary Table 7

Supplementary Table 8

Supplementary Table 9

Supplementary Table 10

Supplementary Table 11

Supplementary Table 12

Supplementary Table 13

Supplementary Table 14

Supplementary Table 15
