# Supplementary material for: A Single Bout of Electroacupuncture Remodels Epigenetic and Transcriptional Changes in Adipose Tissue in Polycystic Ovary Syndrome
Source: Sci Rep. 2018 Jan 30;8:1878. doi: 10.1038/s41598-017-17919-5 (PMC5790004; doi:10.1038/s41598-017-17919-5)
Supplement: Supplementary file 1 — Supplementary info [file 41598_2017_17919_MOESM1_ESM.pdf]

A Single Bout of Electroacupuncture Remodels Epigenetic and Transcriptional Changes in Adipose Tissue in Polycystic Ovary Syndrome

Milana Kokosar[1], Anna Benrick[1,2], Alexander Perfilyev[3], Emma Nilsson[3], Thomas Källman[4], Claes Ohlsson[5], Charlotte Ling[3], Elisabet Stener-Victorin[6]*

[1] Department of Physiology, Institute of Neuroscience and Physiology, Sahlgrenska Academy, University of Gothenburg, Gothenburg, Sweden

[2] School of Health and Education, University of Skövde, Sweden

[3] Epigenetics and Diabetes, Department of Clinical Sciences, Lund University Diabetes Centre, Lund University, Clinical Research Centre, Malmö, Sweden

[4] Department of Medical Biochemistry and Microbiology, NBIS - National Bioinformatics Infrastructure Sweden, SciLifeLab, Uppsala University, Uppsala, Sweden

[5] Centre for Bone and Arthritis Research, Department of Internal Medicine and Clinical Nutrition, Institute of Medicine, Sahlgrenska Academy, University of Gothenburg, Gothenburg, Sweden

[6] Department of Physiology and Pharmacology, Karolinska Institutet, 17177 Stockholm, Sweden

Supplementary Information

Supplementary Table 1: Changes in adipose tissue genes expression from before to after a single bout of electroacupuncture in women with PCOS (n = 21), Q < 0.05. Sorted in alphabetic order.

Supplementary Table 2: Overlap between all genes that changed by a single bout of electroacupuncture (Q < 0.05) and genes that were differentially expressed between cases and controls (Q < 0.05) sorted in alphabetic order.

Supplementary Table 3: Overlap between genes with reversed expression by a single bout of electroacupuncture and genes differentially expressed between cases and controls (Q < 0.05).

Supplementary Table 4: Significant genes contributing to Z-score in Ingenuity Pathway Analysis for expression pathways that are up-regulated by a single bout of electroacupuncture in adipose tissue in women with PCOS.

Supplementary Table5: Significant genes contributing to Z-score in Ingenuity Pathway Analysis for expression pathways that are down-regulated by a single bout of electroacupuncture in adipose tissue in women with PCOS.

Supplementary Table 6: Genes previously linked to insulin resistance, type 2 diabetes and obesity in published genome-wide association studies with corresponding change in gene expression in adipose tissue in women with PCOS after a single one bout of electrocupuncture.

Supplementary Table 7: Changes in DNA methylation in adipose tissue in women with PCOS before versus after a single bout of electroacupuncture (P < 0.05).

Supplementary Table 8: Changes in DNA methylation in adipose tissue in women with PCOS before versus after a single bout of electroacupuncture with a Q = 0.11.

Supplementary Table 9: Changes in DNA methylation in adipose tissue after a single bout of electroacupuncture based on the cross- reactive probes Q < 0.23.

Supplementary Table 10: Genes previously linked to insulin resistance, type 2 diabetes and obesity in published genome wide association studies with corresponding change in methylation in adipose tissue in women with PCOS after a single bout of electrocupuncture.

Supplementary Table 11: Overlap between changes in gene expression (n = 21, Q < 0.05) and changes in DNA methylation (n = 16, Q = 0.11) after single bout of electroacupuncture in adipose tissue from women with PCOS.

Supplementary Table 12: Correlations between changes in glucose disposal rate and top 25 hypermethylated genes and their corresponding probes in response to a single bout of electroacupuncture Q < 11% in 16 women with PCOS.

Supplementary Table 13: Correlations between changes in glucose disposal rate and top 25 hypomethylated genes and their corresponding probes in response to a single bout of electroacupuncture (Q < 11% in 16 women with PCOS).

Supplementary Table 14: Correlations between gene expression (*Q* < 0.05) and DNA methylation (*Q*= 11).

Supplementary Table 15: Genes to be analyzed in inguinal fat in rat by RT-qPCR were selected among genes with largest change in gene expression (Q < 0.05) in response to electroacupuncture in humans.
